# Reply: Multivitamin use may lower risk of preeclampsia: A meta‐analysis

**DOI:** 10.1111/aogs.14417

**Published:** 2022-07-12

**Authors:** Cecilie Holm Christiansen, Stinne Høgh, Line Rode, Jeppe Bennekou Schroll, Hanne Kristine Hegaard, Hanne Trap Wolf

**Affiliations:** ^1^ The Interdisciplinary Research Unit of Women's, Children's and Families' Health Copenhagen University Hospital Rigshospitalet Copenhagen Denmark; ^2^ Department of Obstetrics, Juliane Marie Center Copenhagen University Hospital – Rigshospitalet Copenhagen Denmark; ^3^ Department of Clinical Biochemistry Copenhagen University Hospital ‐ Rigshospitalet – Glostrup Glostrup Denmark; ^4^ Center of Fetal Medicine and Pregnancy, Department of Obstetrics Copenhagen University Hospital – Rigshospitalet Copenhagen Denmark; ^5^ Department of Gynecology and Obstetrics Copenhagen University Hospital ‐ Hvidovre Hospital Hvidovre Denmark; ^6^ Department of Clinical Medicine, Faculty of Health and Medical Sciences University of Copenhagen Copenhagen Denmark

*Sir*,

This is a reply to the comment by Lo and Lo^1^ on our recently published study *Multivitamin use and the risk of preeclampsia: a systematic review and meta‐analysis*.^2^ We would like to thank the authors for showing interest in our research. Our study investigated the association between multivitamin use and the risk of preeclampsia. The data from the observational studies were pooled in a meta‐analysis, showing no significant decrease in the risk of preeclampsia (relative risk 0.85, 95% confidence interval 0.69–1.03), whereas the randomized studies, which were assessed individually, indicated a decreased risk of preeclampsia. Overall, we found very weak evidence that multivitamin use might reduce the risk of preeclampsia. Therefore, we cautiously concluded that the effect of multivitamins on preeclampsia risk remains unclear.

Due to the limited evidence base, Lo and Lo suggest that our review should have included studies investigating fortified food. In our systematic review protocol that was prospectively registered in PROSPERO (registration no. CRD42021214153) prior to the literature search, we clearly indicated that we would only include studies with multivitamins taken as tablets and capsules. We hypothesized that including fortified food could potentially lead to a significant risk of bias due to the difficulties in measuring the actual daily intake of a given food. Hence, fortified foods were not included. In the light of a surprisingly limited evidence base, we agree that it could be interesting to synthesize the evidence regarding fortified food intake and the risk of preeclampsia in future reviews.

Lo and Lo question the exclusion of a randomized study by Chen et al.^3^ We defined our main outcome preeclampsia in PROSPERO as a pregnancy‐induced hypertensive disorder occurring after 20 weeks of gestation characterized by high blood pressure and proteinuria and/or signs of organ dysfunction. Chen et al. included pregnancy‐induced hypertension (without proteinuria) and severe pregnancy‐induced hypertension, the latter defined as the presence of systolic blood pressure ≥160 mm Hg or diastolic blood pressure ≥110 mm Hg.^3^ There seem to be significant differences between preeclampsia and pregnancy‐induced hypertension with respect to their epidemiologic, pathologic, pathogenetic and hemodynamic characteristics.^4^ Hence, we strictly followed our PROSPERO protocol, therefore excluding the study by Chen et al., as the main outcome of their study was pregnancy‐induced hypertension and not preeclampsia.

In the meta‐analysis of adjusted observational data, we excluded the data from Catov et al. due to their use of a hazard ratio instead of an odds ratio.^5^ It can be discussed whether hazard ratios and odds ratios are interchangeable. Additional analyses, including subgroup analyses on body mass index suggested by Lo and Lo, were considered initially, and we agree that subgroup analyses could potentially add interesting information. However, considering the small number of studies and substantial clinical heterogeneity, we ended up excluding such analyses during the review process.

In conclusion, the discussions above show that the GRADE approach is a valuable tool when doing systematic reviews. Despite some disagreements, the conclusion remains the same. The evidence that multivitamin reduces the risk of preeclampsia is low or very low.
